# Enhancing psychosocial care at end of life: A novel simulation training program

**DOI:** 10.1017/S1478951525101430

**Published:** 2026-04-08

**Authors:** Emilia Crnjak, Michelle Kerns, Mariah Stevens, Brianna O'Connell, Lauren Mednick

**Affiliations:** 1 Child Life Services Department, Boston Children’s Hospital, Boston, MA, USA; 2 Immersive Design Systems, Boston Children’s Hospital, Boston, MA, USA; 3 Department of Psychiatry, Boston Children’s Hospital, Boston, MA, USA; 4 Department of Surgery, Boston Children’s Hospital, Boston, MA, USA

**Keywords:** Simulation, training, end-of-life care, child life specialists, staff education, bereavement

## Abstract

**Objectives:**

Providing psychosocial support to pediatric patients and their families at the end of life represents one of the most challenging yet vital aspects of healthcare practice. Despite the presence of grief and loss training in many pediatric healthcare professionals’ educational backgrounds, opportunities for practical training experience in delivering end-of-life care remain limited. This study explored the use of simulation-based training to enhance the self-reported knowledge, skills, and comfort levels of child life specialists in providing psychosocial care during end-of-life situations.

**Methods:**

Forty-three child life specialists participated in the simulation-based training, which was combined with traditional didactic instruction, and the associated research study. Pre- and post-training surveys were used to assess impact of the training on child life specialists’ self-reported knowledge of end-of-life care and comfort in providing this care.

**Results:**

A statistically significant increase was seen in all measured aspects of self-reported knowledge and comfort in providing end-of-life care following the training.

**Significance of results:**

Simulation combined with traditional instruction methods provides an effective way to train healthcare professionals in providing high-stakes psychosocial care while protecting patients and families from the added strain of trainees and excess staff presence during sensitive times.

## Introduction

The death of a child is one of the most difficult life circumstances that a family can face, and the contexts surrounding the death, including the support received, can have a profound impact on long-term coping with this loss (Hasdenteufel and Quintard 2022). Families of adult patients have reported feeling unsupported and uninvolved in the moments during and after their loved one’s death (Nelson et al. 2010). Similarly, parents of pediatric patients have reported feeling the loss of their role as parent and uncertainty in what to do during the time of their child’s death in a hospital environment (Butler et al. 2019). When surveyed, parents have reported benefiting from specific support from medical staff, including modifications to de-medicalize the environment, streamlined communication, logistical support, and offering mementos such as photographs of the child (Butler et al. 2019).

Supporting children and families during end of life is an aspect of care that every pediatric healthcare professional should have some degree of confidence and competence in, regardless of area of specialty. Yet, many healthcare professionals report a sense of unease or inexperience related to the conversations and support that is provided during these most sensitive times (Contro 2004). Numerous factors, such as clinicians’ own discomfort with death and dying, as well as limited training opportunities, can lead to feelings of doubt and uncertainty in one’s abilities to support bereaved families (Woo et al. 2006). Oftentimes, healthcare professionals are faced with a fear of not knowing what to say during moments of such immense grief, but when medicine or surgery can no longer heal, all that is left are words and presence in the face of grief and loss.

Certified child life specialists (CCLSs) are members of the healthcare team whose specialized background in child development and psychosocial care can equip them with the ability to support children and families during end of life (Boles et al. 2020). In the context of end-of-life and bereavement care, CCLSs may provide emotional support to the family, aid in modifying the hospital room for comfort, help with legacy projects, and offer age-appropriate support to siblings, all of which decrease the risk of complicated mourning and improve family coping (Basak et al. 2019). With the support from child life specialists, caregivers have expressed positive feedback from their experiences in working with both their dying child and siblings to optimize coping and grief support (Boles et al. 2020).

However, despite taking necessary coursework in bereavement and completing internship hours to obtain certification, many CCLSs enter the field with little direct experience in end-of-life care, limiting their ability to translate this conceptual knowledge into practice with confidence. Similarly, other health professionals, including physicians, also report limited training and experience in supporting families in end-of-life care (Sikstrom et al. 2019). With all of these healthcare professionals, much of this lack in training and experience is likely due to the relatively infrequent occurrence of pediatric death in many healthcare settings.

In many areas of medicine, simulation-based education (SBE) is commonly used to provide opportunities for repeated practice of medical scenarios within a safe, controlled environment in order to improve knowledge, skill acquisition, and confidence and is often used to practice responses to low frequency events. This style of training has been shown to increase self-perceived competence and confidence during high-stress events such as a medical code (Allan et al. 2010), difficult conversations with patients and families (Paparo et al. 2021), and mental health assessments (Piette et al. 2018). SBE has specifically been used to increase confidence and skills in end-of-life care for new PICU nurses (Hillier et al. 2022). Studies have further concluded that integrating simulation-based training with traditional instructional methods results in significantly improved clinical skill acquisition and long-term retention (Ajemba et al. 2024) and effectively improves both technical and non-technical (human factor) skills in qualified healthcare teams (Abildgren et al. 2022).

Taken together, in an effort to address the gap in training and experience and increase self-reported knowledge and comfort in providing end-of-life support to families, we created an innovative and comprehensive SBE which was then offered to all CCLSs at our large metropolitan children’s hospital. The current study aims to evaluate the effectiveness of combining SBE with didactic instruction to increase self-reported knowledge and confidence in providing end-of-life care to families.

## Methods

### Course development

A committee of 3 CCLSs with expertise in bereavement was formed to address this notable gap in comfort levels and knowledge related to providing end-of-life care. A needs assessment survey consisting of 25 questions was conducted via REDCap (Research Electronic Data Capture) and emailed to all child life specialists in the department. REDCap is a secure, web-based software platform designed to support data capture for research studies (Harris et al. 2009, 2019). The survey included a combination of multiple choice, Likert scale, and open-ended questions exploring participants’ primary units and populations served, years of experience, self-perceived knowledge and comfort levels in end-of-life care, and perceived needs for further training and support.

Out of 57 CCLSs employed at the hospital at the time of the needs assessment, 43 CCLSs (75%) completed the survey. Participants indicated self-perceived lack of knowledge, overall discomfort, and desire for more hands-on practice or shadowing experience with bereavement care. Specifically, only 28% of CCLSs reported feeling very knowledgeable and only 19% reported feeling very comfortable providing support to families surrounding the death of a child.

In an effort to address these reported concerns, this committee, in collaboration with the hospital’s robust simulation program, utilized their expert experience and practice within this hospital, as well as information gathered from a literature review on interventions that families have found meaningful and supportive (Butler et al. 2019; Boles et al. 2020), to create the current course.

### Course description

The course centers around 5 core elements of child life interventions at end of life: emotional support, environmental support, sibling support, memory making/legacy projects, and specific support offered to the dying patient when alert and aware (Goodhue 2016). To foster participants’ comfort level and confidence in their skills prior to engaging in realistic simulation scenarios, the course began with a 1-hour didactic teaching session focusing on these core interventions. Facilitators then guided participants through a 45-minute memory making practice session. This activity was considered a low-fidelity simulation as it did not take place in a realistic setting. Specifically, participants practiced using each other and facilitators’ hands as models and were guided in real-time by facilitators offering instruction and suggestions. Finally, participants engaged in 3 high-fidelity simulated end-of-life scenarios, which took place in a realistic hospital room setting with simulated patients in the form of mannequins and actors portraying caregivers. These scenarios were run in a realistic manner with no input from facilitators or pauses to the scenario.

The high-fidelity scenarios were based on real case scenarios that CCLS facilitators had experienced in their own work; however, family demographics and detailed information were adjusted. Three scenarios covered chronic illness, trauma, and an unfavorable surgical outcome. Learning objectives included introduction of the child life role in the various contexts, sibling support, and memory making (see Appendix A for more details). All participants were given a brief introduction to the scenario, including family demographics and current plan of care. Facilitators assigned 2 participants at a time to engage in each scenario while the remaining participants observed via video feed to mitigate crowding in the simulated hospital room and preserve fidelity.

After each scenario, a facilitator trained in simulation debriefing methods led a structured debrief with the entire learning group, including simulation participants, observing participants, and actors, allowing participants to process their emotional reactions, summarize the scenario, and analyze the impact of interventions used during the simulation.

### Participants

Over a 2-year period, the course was run 9 times with 4–6 child life specialists participating in each session, combined for a total of 48 participants. Of the 48 participants, many of these participants also completed the needs assessment. However, due to staff turnover in the department, not all individuals who were represented in the needs assessment were also represented in the research data.

Participants for each training group were carefully selected to reflect a wide range of bereavement experience ranging from novice to seasoned, in order to enhance opportunities for emotional safety and peer-to-peer cohort learning. Of the 48 course participants, 46 consented to the associated research study and completed at least the pre-participation survey.

Twenty-two percent of the research participants had worked in the child life field for less than 1 year, 22% for 1–4 years, 37% for 5–9 years, and 30% for 10 years or longer. Thirty percent of participants reported regularly providing end-of-life care, and an additional 15% reported providing back-up coverage to areas where they may be asked to provide end-of-life care.

During the previous year, 32% reported no involvement in end-of-life cases, 34% reported involvement in 1–5 cases, 21.7% reported involvement in 5–14 cases, and 10.9% reported involvement in 15 or more end-of-life cases (see Table 1). The child life department demographics at the time of this study was all female and predominantly white.

**Table 1.** Participant demographics

**Years as a practicing child life specialist**				
	**Pre-participation (*n* = 46)**	**Post-participation (*n* = 39)**	**3-month follow-up (*n* = 23)**	**6-month follow-up (*n* = 27)**
<1 year	10	10	4	7
1–4 years	17	14	9	12
5–9 years	5	4	2	2
10+ years	14	11	8	6

**Self-reported number of end-of-life cases involved in**				
	Pre-participation* (*n* = 46)	Post-participation* (*n* = 39)	**3-month follow-up^+^ (*n* = 23)**	**6-month follow-up^+^ (*n* = 27)**
None	15	14	15	13
<5	16	13	7	10
5–14	10	8	1	4
15+	5	4	0	0

* Year prior to training.

+ Since training.

## Measures

Participants were asked to complete surveys before, after, and at 3- and 6-month intervals after course completion (see Table 1 for descriptive details of participants at each time point). Study data were collected and managed using REDCap. Pre-course surveys included background questions, as well as a 10-item questionnaire created for this study that asked participants to rate both their self-perceived knowledge and comfort level with key psychosocial interventions using a 5-point Likert-scale ranging from 1: not at all comfortable/knowledgeable to 5: very comfortable/knowledgeable. This 10-item questionnaire was then repeated in the post-course survey. This survey also included the opportunity to provide qualitative feedback on what aspects of the training were most helpful and whether there were topics participants would have liked more focus on.

The 3- and 6-month follow-up surveys evaluated how much the course was perceived to have affected participants’ knowledge and comfort levels during subsequent end-of-life work, if they had participated in any since the training. At 6 months, those who had not participated in any end-of-life work were asked if they felt the training would have an impact should they be called to participate in an end-of-life case.

## Results

The Wilcoxon signed-rank test was used to examine differences in questionnaire items between the pre-participation and post-participation surveys. All comparisons were significant at *P* < .001 (see Table 2), indicating improved self-perceived knowledge and comfort in all areas after completing the course.

**Table 2.** Pre-participation and post-participation surveys

		*n*	*Z* value	*P* value	Mean pre-participation	Mean post- participation
Providing emotional support to families	Knowledge	39	−4.886	<.001	3.39	4.41
	Comfort	39	−4.167	<.001	3.43	4.18
Providing environmental support to families	Knowledge	39	−4.391	<.001	3.61	4.51
	Comfort	39	−4.092	<.001	3.52	4.36
Introducing memory making activities to families	Knowledge	38	−4.582	<.001	3.47	4.41
	Comfort	38	−4.278	<.001	3.42	4.26
Participating in memory making activities with families	Knowledge	38	−4.877	<.001	3.41	4.53
	Comfort	38	−5.352	<.001	3.46	4.26
Providing information to caregivers on supporting siblings	Knowledge	39	−4.513	<.001	3.54	4.41
	Comfort	39	−4.204	<.001	3.41	4.15
Talking to siblings about death	Knowledge	39	−4.567	<.001	3.3	4.26
	Comfort	39	−4.251	<.001	3.18	3.9
Participating in memory making with siblings	Knowledge	39	−4.556	<.001	3.47	4.46
	Comfort	39	−4.007	<.001	3.52	4.21
Talking to parents about the dying child’s understanding and experience of death	Knowledge	39	−4.94	<.001	2.8	4.13
	Comfort	39	−4.722	<.001	2.67	3.79
Talking to the dying child about death	Knowledge	39	−5.225	<.001	2.46	3.77
	Comfort	38	−4.651	<.001	2.39	3.45
Confidence in providing end-of-life care		37	−4.323	<.001	3.22	4.16

To assess whether changes in self-perceived knowledge and comfort differed by years of clinical experience, we computed change scores (post minus pre) for each variable, graphed these across 4 experience categories (<1 year, 1–4 years, 5–9 years, 10+ years), and tested for group differences using Kruskal–Wallis tests. Changes in knowledge and comfort scores did not significantly differ by years of clinical experience (all Kruskal–Wallis *p*’s > .05; see Table 3).

**Table 3.** Change from pre- to post-survey by years of clinical experience

		Chi-square	*df*	*P* value
Providing emotional support to families	Knowledge	1.631	3	0.65
	Comfort	4.612	3	0.2
Providing environmental support to families	Knowledge	1.295	3	0.73
	Comfort	0.569	3	0.9
Introducing memory making activities to families	Knowledge	0.841	3	0.84
	Comfort	0.88	3	0.83
Participating in memory making activities with families	Knowledge	0.872	3	0.83
	Comfort	5.818	3	0.12
Providing information to caregivers on supporting siblings	Knowledge	2.392	3	0.5
	Comfort	3.787	3	0.29
Talking to siblings about death	Knowledge	2.747	3	0.43
	Comfort	2.024	3	0.57
Participating in memory making with siblings	Knowledge	0.228	3	0.97
	Comfort	4.294	3	0.23
Talking to parents about the dying child’s understanding and experience of death	Knowledge	1.812	3	0.61
	Comfort	4.815	3	0.19
Talking to the dying child about death	Knowledge	2.735	3	0.43
	Comfort	6.109	3	0.11

Twenty-three participants completed the 3-month follow-up survey and 30% (*n* = 7) of these respondents had participated in at least 1 end-of-life case. All of those participants who had participated in end-of-life care said that the course had at least some impact on their knowledge of what interventions to provide and comfort in providing interventions during those cases.

Twenty-seven participants completed the 6-month follow-up survey and 52% (*n* = 14) of this group had participated in at least 1 end-of-life case. All participants found the training to have had at least some impact on their knowledge, and 93% found the training to have had at least some impact on their comfort during those cases. Of those who had not participated in an end-of-life case after 6 months (*n* = 13), all answered that they believed the course would have at least some impact on their knowledge of what interventions to offer and comfort in providing those interventions if they were to be called to support an end-of-life case (see Table 4).

**Table 4.** Three- and six-month follow-up surveys

**Since the time you participated in the training, how many end-of-life cases have you participated in?**					
		0 Cases	1 Case	2–4 Cases	5+ Cases
3-month follow-up (*n* = 23)		15 (65.2%)	3 (13%)	4 (17.4%)	1 (4.3%)
6-month follow-up (*n* = 27)		13 (48.1%)	2 (7.4%)	8 (29.6%)	4 (14.8%)

How much do you think this training impacted your knowledge and comfort?*					
		No impact	Very little impact	Some impact	Significant impact
3-month follow-up (*n* = 8)	Knowledge	0 (0%)	1 (12.5%)	3 (37.5%)	4 (50%)
	Comfort	0 (0%)	0 (0%)	4 (50%)	5 (50%)
6-month follow-up (*n* = 14)	Knowledge	0 (0%)	0 (0%)	8 (57.1%)	6 (42.9%)
	Comfort	0 (0%)	1 (7.1%)	6 (42.9%)	7 (50%)

**If you were called upon to participate in an end-of-life case now, do you think that your participation in the training 6 months ago would have an impact on your knowledge and comfort?^+^**					
		No impact	Very little impact	Some impact	Significant impact
6-month follow-up (*n* = 13)	Knowledge	0 (0%)	0 (0%)	8 (61.5%)	5 (38.5%)
	Comfort	0 (0%)	0 (0%)	11 (84.6%)	2 (15.4%)

* Asked only of those who have participated in at least 1 end-of-life case.

+ Asked only of those who had not participated in any end-of-life cases at 6-month follow-up.

To interpret qualitative data, responses to a free text question asking participants what aspects of the training they felt were most helpful were independently categorized into themes by 3 researchers. Categorizations were then compared and agreement was reached for all categories. Four themes were determined from the free text response to be most helpful: simulation combined with traditional didactic instruction, simulation, specific tips, and peer-to-peer learning. Most significantly, 46% of participants identified SBE being most helpful to their learning and 28% specifically stated that the combination of the traditional teaching methods and simulation aspects were most helpful. In addition, despite the heavy subject matter and pressure of performing the simulation scenarios, participants expressed a sense of comfort and safety in the training environment that fostered productive learning opportunities. (see Tables 5 and 6).

**Table 5.** Thematic analysis of qualitative data

What did you find most useful/helpful about this training?		
Theme	Responses	Percent of responses *n* = 39
Simulation combined with traditional instruction methods	11	28%
Simulation	18	46%
Specific tips	5	13%
Peer-to-peer learning	6	15%

**Table 6.** Selected responses

What did you find the most useful/helpful about this training?
“Despite how nerve wracking it was, I believe the most useful component of the training were the actual simulation scenarios. Being put into a real life scenario, alongside extremely devoted and talented actors was the ideal way to practice bereavement and end of life care which was something I have been really looking for in my child life career thus far. I also really appreciated the opportunity to practice memory making on both myself and my peers to understand both perspectives.”
“I really appreciated the format of the training … having the didactic piece first, then the hands-on practice with the EOL materials, and then the practice scenarios/simulations with the actors. The actors/scenarios felt so realistic and it was very unique to have the opportunity to practice scenarios outside of our comfort zones in a very safe and supportive environment. I have never had experience working in the ED or with EOL situations involving suicide. It really stretched me and gave me confidence that if I were ever to be placed in a situation like this, I would feel better prepared.”
“I believe the training’s agenda/schedule set up was really helpful. I liked how we first started with an overview of supporting children and families with end of life, then we were able to practice memory making with others in our group and then participate in end life scenarios with actors. The training provided us with hands on experiences which gave us an opportunity to know some of the emotions we and others might feel during an end of life moment. The training also gave us a safe space to learn from each others experiences and ask questions about end of life.”
“The flow of the training was helpful as we had the opportunity to review material then practice it in the simulation. The actors and SIM space were fantastic and really helped to make the situation feel real.”
“The opportunity to practice in a low-stakes setting, while still feeling very realistic.”

## Discussion

The significant increase in self-perceived knowledge and comfort levels in all measured aspects of end-of-life care, along with the feedback regarding the multiple modes of learning, suggests that this combination of simulation and traditional instruction is an effective method of training healthcare professionals in high-stakes psychosocial care such as bereavement.

Additionally, similar increases seen in both novice and experienced clinicians’ knowledge and comfort level suggests that simulation-based training can be utilized for continued education and professional development and should not be limited to new staff orientation or pre-professional education. This is supported by previous research findings that simulation can be effectively utilized to increase knowledge and skills for both healthcare practitioners as well as medical trainees (Aggarwal et al. 2010). The follow-up surveys at 3- and 6-month intervals also indicate lasting effects of this training, suggesting that simulation is a high-impact way to not only train infrequently used skills but to retain those skills. This finding is consistent with previous research which found that medical residents who participated in a simulation-based training for Advanced Cardiac Life Support (ACLS) demonstrated greater skill retention at 6 and 14 months post-training compared to the control group (Wayne et al. 2006).

Although many participants expressed benefits and increased comfort after the training, it was not possible to represent every important bereavement skill within the 3 simulated scenes. Future training could include other topics that were mentioned in the qualitative feedback, such as death by suicide, which was not covered due to time constraints. Participants also mentioned wanting more practice with talking to children about their own serious medical condition and death; however, there are ethical implications for involving a child actor in these scenarios, as this could potentially impact their emotional well-being.

Further limitations to this study include the absence of certain demographic details about the study participants (e.g., ethnicity) and having limited diversity in other demographic variables, which limit the generalizability of the findings. Future research could examine the impact on more diverse groups, including other medical professions. In addition, the decreased response rate at 3 and 6 months limits our ability to fully understand the long-term impact of the training.

Finally, while the simulation training model described in this manuscript utilized substantial resources, including a fully equipped simulation lab and trained actors, it is important to note that its core principles can be adapted for hospitals with fewer resources. For instance, smaller hospitals could utilize lower-cost simulation tools, such as role-playing exercises with staff members instead of professional actors. By leveraging existing spaces within the hospital, these institutions can minimize the financial investment required.

Additionally, collaboration with other healthcare facilities or educational institutions may provide access to shared resources, further enhancing the accessibility of such training programs. The key to replicating this model lies in flexibility and creativity in utilizing available resources, while maintaining the integrity and effectiveness of the training objectives.

## Conclusion

The impact of psychosocial simulation training combined with traditional instruction methods within the realm of end-of-life care is significant. Psychosocial simulation offers a valuable opportunity for healthcare professionals providing grief and bereavement care to confidently build their skills to best support patients and families during these times. Providing a safe space to explore and understand the complexities of grief through didactic teaching and fostering opportunities to practice skills through simulation equips professionals with the necessary skills and resilience to navigate the emotional challenges inherent in end-of-life care, ensuring that the best possible support is given to families during their most fragile of times.

## Appendix

**Simulation Scenarios**

**Scenario 1: Sibling Support – Preparing for Goodbye**

**Learning Objectives**:

1. Introduce and discuss sibling support at end-of life with parents
2. Coach parents on how to communicate with a sibling to prepare for a visit/end of life of sibling
3. Provide resources to optimize sibling coping

**Patient Details:**

- **Name:** Julia Martin, 6 years old
- **Diagnosis**: Acute lymphoblastic leukemia (ALL), relapsed after bone marrow transplant
- **Family Context**:
  - Parents: John (landscaping business owner) and Joanne (stay-at-home parent)
  - Sibling: Jack, 8 years old male
  - Family culture: Strong Catholic faith and community support
  - Case Information: Julia was made a DNR/DNI overnight and that parents are wondering if now is the time to bring their 8-year-old son Jack in to visit as she is progressing quickly. They have been offered sibling support by their primary CLS but were not ready to engage at the time. Jack is aware that she is very sick, but not that she is dying. Parents ask: “do we tell him that this is goodbye?” and “how would we even begin to tell him that?”

**Scenario 2: Memory Making with Sibling**

**Learning Objectives**:

1. Introduce memory-making and the role of child life
2. Facilitate memory-making activities at the bedside
3. Support sibling engagement during memory-making and navigate family dynamics

**Patient Details:**

- **Name:** Sarah Wilson, 18 years old
- **Diagnosis**: Severe head trauma from a car accident; brain death testing pending
- **Family Context**:
  - Parents: Veronica (bartender) and Sergei Sr. (long-haul truck driver)
  - Sibling: Sergei Jr., 16 years old male
  - Family Culture: Tight knit immediate family. Sergei and Sarah bond with quality time playing sports together.
  - Case Information: Mom (Veronica) and brother (Sergei Jr.) are at bedside. Veronica shares that Sergei isn’t interacting with Sarah, and she isn’t sure how to help him say goodbye. Parent requests that child life check in with Sergei Jr. and begin memory making projects with him. Sergei Jr. presents as withdrawn and disengaged.

**Scenario 3: Advanced Memory Making & Sibling Support/Advocacy**

**Learning Objectives**:

1. Introduce the role of child life and build rapport in sensitive situations
2. Provide emotional support and advocacy for sibling involvement
3. Offer guidance on discussing end of life with a young sibling and preparing for a visit

**Patient Details:**

- **Name:** Grace O’Connor, 1 week old female
- **Diagnosis**: Biliary atresia, end-stage liver failure
- **Family Context**:
  - Parents: Shauna (Lawyer) and James (Financial Advisor)
  - Sibling: Hazel, 3 years old female
  - Family Culture: Glazes over things, sweeps hard things under the rug to keep up with appearances, strives for “perfection.”

- Case Information: Shauna doesn’t know if she should tell Hazel about Grace dying because in her view, “Hazel is too young to understand and hasn’t even met Grace” and she doesn’t want to “traumatize” her daughter by bringing her in to say goodbye to Grace. She is crying but slowly able to gather herself together enough to engage with enough validation and emotional support.

James appears very stoic and is trying to be “strong” for his wife by comforting her and holding her. He also is hesitant to bring in Hazel but is open to talking about how they would tell her and what that would look like if they do bring Hazel in. He seeks guidance from the CLS asking, “how do we explain this to Hazel? How do we tell her that Grace is dying? How do we support her in saying goodbye?”
